# Compositional Effects on the Performance of High-Permeability Emulsified Asphalt for Prime Coat Applications

**DOI:** 10.3390/ma18184430

**Published:** 2025-09-22

**Authors:** Zhen Qin, Xiang Liu, Shaopeng Zheng, Simiao Pan, Xiaolong Li, Jingpeng Jia, Hang Xiong

**Affiliations:** 1 Faculty of Transportation Engineering, Kunming University of Science and Technology, Kunming 650500, China; zhen@stu.kust.edu.cn (Z.Q.); pansimiao@stu.kust.edu.cn (S.P.); xt15519771782@outlook.com (H.X.); 2 School of Highway, Chang’an University, Xi’an 710061, China; 3 Yunnan Science & Technology Research Institute of Highway, Kunming 650051, China; xiaolongli198909@163.com (X.L.); 13700692983@163.com (J.J.); 4 Broadvision Engineering Consultants, Kunming 650011, China; 5 Yunnan Digital Technology Innovation Centre of Modern Integrated Transportation, Kunming 650051, China

**Keywords:** high permeability, emulsified asphalt, kerosene, emulsifier, grey relational analysis

## Abstract

High-permeability emulsified asphalt has emerged as a promising prime coat for enhancing interlayer bonding in semi-rigid pavement structures. However, its widespread adoption remains limited by insufficient permeability and inconsistent mechanical properties. This study systematically investigated the effects of emulsifier ionic type (cationic or anionic), kerosene dosage (0–20%), and diluted asphalt content (corresponding to oil-water ratios of 5:5 and 4:6) on the comprehensive performance of high-permeability emulsified asphalt. Fundamental physical tests (sieve residue, evaporation residue, penetration, softening point, ductility), permeability evaluation, rotational viscosity measurements, and adhesion performance tests were conducted. Grey relational analysis (GRA) was employed to quantify the influence of each factor and their interactions on key performance metrics. The results reveal that anionic emulsifiers significantly improved low-temperature ductility and permeability. A low kerosene dosage (<10%) enhanced permeability and viscosity but compromised thermal stability at higher levels. Reducing the diluted asphalt content partially offset these adverse effects. GRA identified kerosene dosage as the dominant factor influencing permeability, softening point, and adhesion performance while emulsifier ionic type primarily affected ductility, and oil-water ratio strongly governed emulsification quality and viscosity. These findings provide quantitative insights for optimizing the composition of high-permeability emulsified asphalt and serve as a theoretical foundation for its engineering application in durable prime coats.

## 1. Introduction

In semi-rigid base asphalt pavement systems, which are widely employed in Chinese highway construction, the significant difference in mechanical properties between the cement-stabilized base and the asphalt surface layer often leads to poor interlayer compatibility [1]. This mismatch can induce weak bonding at the interface, resulting in delamination, slippage, and other early-stage pavement failures under repeated traffic loads [2,3,4]. To address this issue, prime coats are applied between the base and surface layers to promote interlayer adhesion [5]. A well-designed prime coat penetrates the base surface, forming a thin, cohesive, and water-resistant bonding layer that enhances structural continuity, prevents moisture intrusion, and extends pavement service life.

Among the prime coat materials currently used in pavement construction, kerosene-diluted asphalt and emulsified asphalt are the most prevalent [6]. The former is prepared by blending hot asphalt with kerosene or other petroleum distillates. Upon application, the volatile components evaporate, leaving behind a residue of asphalt binder that exhibits excellent permeability. However, its use is limited by safety concerns, environmental hazards, and poor long-term storage stability due to high volatility [7]. In contrast, emulsified asphalt is produced by mechanically dispersing asphalt into fine droplets within a water-based solution containing emulsifiers, forming a stable oil-in-water or water-in-oil emulsion. Following application, the emulsion undergoes breaking and water evaporation, leaving a cohesive asphalt film on the substrate [7,8,9]. While emulsified asphalt offers notable advantages, such as improved bonding strength, lower environmental impact, and greater storage stability, its insufficient permeability severely impairs its bonding effectiveness in semi-rigid base pavement systems [10,11,12].

To overcome the permeability limitations of conventional emulsified asphalt, high permeability variants have been developed by incorporating composite emulsifier systems and non-volatile light oils into the matrix asphalt [6,7]. The performance of such formulations as prime coats is critically dependent on the material composition and formulation design. Key functional attributes, such as permeability, interlayer adhesion, and emulsion stability, are strongly influenced by the proportions and physicochemical interactions among constituent materials. A well-optimized formulation can markedly improve interlayer bonding, promote adequate permeability and curing of the base surface, mitigate early-stage pavement distresses, and ultimately extend pavement service life. Therefore, systematic investigation into the formulation–performance relationship of high-permeability emulsified asphalt is of substantial engineering and practical significance.

A growing body of research has sought to enhance the permeability of emulsified asphalt by modifying its composition and formulation design parameters. Several studies have explored the use of penetrants, emulsifier dosage adjustment, and particle refinement to improve emulsion infiltration. For example, Song et al. investigated the influence of asphalt content, emulsifier dosage, and grinding duration on the particle size distribution and permeability characteristics of emulsified asphalt. Their findings revealed that reducing both the average particle size and evaporative residue content significantly improved permeability, while also lowering the Engler viscosity [13]. Zhang et al. developed a high-permeability emulsified asphalt through comparative evaluation of five mix formulations and assessed its adhesion, cohesion, and water resistance via interlayer shear and immersion tests, ultimately identifying the optimal blend [6]. Chen et al. demonstrated that incorporating kerosene as a penetrant improved permeability performance, but noted that excessive dosages adversely affected other key properties, suggesting the need for dosage optimization [14]. In addition, recent studies have explored the incorporation of bio-oil from waste sources as a sustainable additive. Shi et al. employed molecular dynamics simulations and maximum packing density tests to evaluate the permeability performance of emulsified asphalt modified with biomass oil, reporting optimal results at a 1:3 bio-oil-to-asphalt ratio [1]. Similarly, Liu et al. observed that permeability depth increased with higher penetrant dosage but decreased with excessive asphalt content when diluted with bio-oil filtrate [5]. These studies collectively underscore the importance of rational formulation design and additive selection in achieving high-performance emulsified asphalt. However, despite these advancements, a comprehensive understanding of how key composition factors, particularly emulsifier ion type, kerosene dosage, and oil-water ratio, quantitatively affect permeability and bonding performance remains limited.

Although prior studies have explored the use of penetrants, emulsifier dosage adjustments, and bio-based modifiers to improve the permeability of emulsified asphalt, the effects of key formulation variables on overall material performance remain insufficiently understood. In particular, the influence of emulsifier ionic type (cationic vs. anionic) on low-temperature flexibility and permeability has not been quantitatively examined. The functional threshold of kerosene dosage—beyond which performance deterioration occurs—also lacks systematic verification. Moreover, the effect of diluted asphalt content (i.e., oil-water ratio) on properties such as evaporative residue, viscosity, and permeability has not been comprehensively evaluated. Existing studies typically focus on single-factor variation, neglecting the potential interactions among multiple variables. These gaps underscore the need for a multi-factorial experimental study to clarify the individual and combined influences of emulsifier type, kerosene dosage, and oil-water ratio on the fundamental properties of high-permeability emulsified asphalt.

Therefore, the present study quantitatively investigated the effects of emulsifier ion type, kerosene dosage, and oil-water ratio on the emulsification characteristics of high-permeability emulsified asphalt. The comprehensive performance of the asphalt was evaluated using fundamental physical property (sieve residue, evaporation residue content, penetration, softening point, and ductility) tests, permeability tests, rotational viscosity measurements, and adhesion performance test. In addition, grey relational analysis was employed to rank the influence of different formulation design factors on various performance indicators. The analysis identified the priority components that should be adjusted when formulating emulsified asphalt with specific performance requirements. This study provides a theoretical basis for the quantitative formulation and formulation design optimization of high-permeability emulsified asphalt.

## 2. Materials and Methods

### 2.1. Raw Materials

The high-permeability emulsified asphalt formulations in this study were prepared using matrix asphalt, water, emulsifiers, penetrants, and pH-adjusting additives.

#### 2.1.1. Matix Asphalt

Gaofu 70# asphalt produced by Sinopec Maoming Petrochemical Co., Ltd. (Maoming, China) was selected as the matrix asphalt [15,16], The basic parameters of the matrix asphalt are detailed in Table 1.

#### 2.1.2. Emulsifiers

In emulsified asphalt systems, water acts as the continuous phase, while asphalt droplets form the dispersed phase. To ensure emulsion stability, emulsifiers are necessary to prevent phase separation. Given that single emulsifiers often fail to meet performance requirements, a blend of emulsifiers with different ionic characteristics was employed. Specifically, cetyltrimethylammonium bromide (CTAB, cationic) and sodium dodecylbenzene sulfonate (SDBS, anionic) were each combined with octyl phenol polyoxyethylene ether (OP-10, non-ionic) to formulate composite emulsifier systems. Sinopharm Chemical Reagent Co., Ltd. (Shanghai, China) produced the CTAB and SDBS, while OP-10 was obtained from Tianjin Kermel Chemical Reagent Co., Ltd. (Tianjin, China) The basic parameters of the emulsifiers are detailed in Table 2.

#### 2.1.3. Penetrants

To enhance permeability, kerosene with a purity above 90% was used as the diluent and polyoxyethylene isooctyl ether (JFC-E) served as the penetrant. The kerosene was supplied by China Petroleum & Chemical Corporation (Beijing, China), and JFC-E was obtained from Shandong Yousuo Chemical Reagent Co., Ltd. (Shandong, China) The basic parameters of the penetrants are detailed in Table 3.

#### 2.1.4. pH-Adjusting Agent

In addition, calcium chloride and hydrochloric acid were used to adjust the pH values of anionic and cationic emulsions, respectively, ensuring formulation stability.

### 2.2. Formulation Design and Preparation of High-Permeability Emulsified Asphalt

In this study, a factorial formulation design was adopted to investigate the effects of key formulation parameters on the performance of high-permeability emulsified asphalt. Three control variables were considered: emulsifier ionic type, kerosene dosage, and oil-water ratio (defined as the mass ratio of diluted asphalt to soap solution). Kerosene dosage was varied at five levels (0%, 5%, 10%, 15%, and 20%), while two emulsifier types (cationic and anionic) and two oil-water ratios (5:5 and 4:6) were selected. A total of 20 distinct formulations were developed, as summarized in Table 4.

In this study, two types of emulsifier systems were designed for the soap solution: cationic and anionic. Each system consisted of a primary emulsifier combined with a nonionic surfactant (OP-10) to enhance emulsion stability. For the cationic system, CTAB was used as the main emulsifier, while SDBS was employed for the anionic system. A penetrant (JFC-E) was incorporated into both systems to improve permeability, and pH-adjusting agents (calcium chloride for the anionic solution and hydrochloric acid for the cationic solution) were added to ensure formulation stability. The detailed composition of each soap solution is summarized in Table 5.

**Figure 1:**
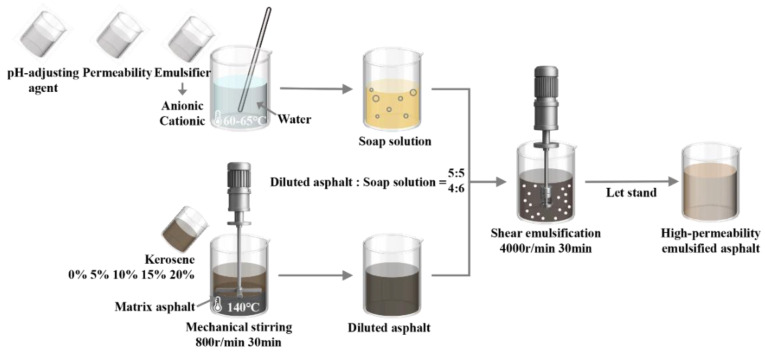
Preparation process of high-permeability emulsified asphalt.

Emulsified asphalt can generally be prepared using either spontaneous emulsification or mechanical dispersion techniques [18,19,20]. Among these, mechanical dispersion is widely adopted in practice due to its superior efficiency, process stability, and scalability. In this study, high-permeability emulsified asphalt was fabricated using a mechanical dispersion method, as depicted in Figure 1. Specifically, predetermined amounts of emulsifier, penetrant, and pH-adjusting agents were mixed into deionized water and maintained at 60–65 °C to form a stable soap solution. Simultaneously, the matrix asphalt was heated to 140 °C, into which the appropriate quantity of kerosene was added under mechanical stirring at 800 rpm for 30 min to obtain the diluted asphalt. The resulting soap solution and diluted asphalt were then blended at 4000 rpm for 30 min using a high-speed homogenizer to produce a uniform emulsified asphalt. After emulsification, the product was allowed to cool naturally to room temperature.

**Figure 2:**
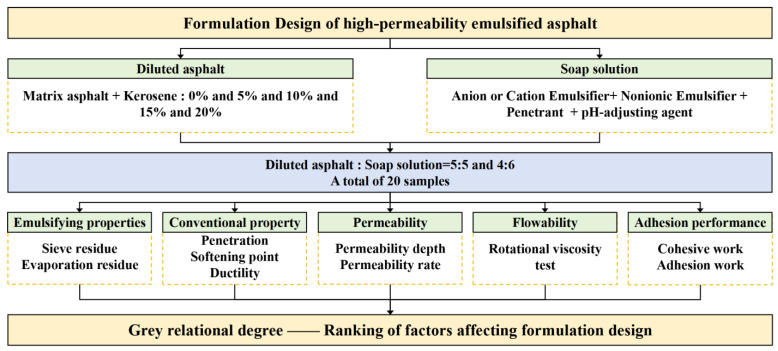
Flowchart of the experimental procedure for this study.

Following the preparation process, the experimental procedure proceeded with systematic performance evaluation and grey relational analysis. A schematic flowchart illustrating the overall research framework, including material selection, formulation matrix design, emulsified asphalt preparation, performance testing, and factor-performance correlation analysis, as presented in Figure 2.

### 2.3. Fundamental Physical Properties

In this study, the fundamental physical properties of the high-permeability emulsified asphalt were evaluated in accordance with the Test Methods of Bitumen and Bituminous Mixtures for Highway Engineering (JTG E20-2011) [21]. The tests included residue on sieve, evaporation residue content, and standard performance indicators of the evaporation residue, including penetration (25 °C, 100 g, 5 s), softening point (°C), and ductility (15 °C, 5 cm/min). The evaporation residue was obtained by subjecting the emulsified asphalt to a controlled heating and evaporation process, following the procedures specified in the standard. The residue on sieve and evaporation residue content were calculated according to Equations (1) and (2), respectively.

(1) $$P_{r}=\frac{m_{2}-m_{1}}{m}\times 100,$$

where $P_{r}$ represents the sieve residue content (%), m is the mass of emulsified asphalt sample (g), $m_{1}$ is the mass of the sieve and metal tray (g), and $m_{2}$ is the total mass of sieve, tray, and residue (g).

(2) $$P_{b}=\frac{m_{3}-m_{1}}{m_{2}-m_{1}}\times 100,$$

where $P_{b}$ represents the evaporation residue content (%), $m_{1}$ is the mass of container and glass rod (g), $m_{2}$ is the total mass of container, rod, and emulsion (g), and $m_{3}$ is the total mass of container, rod, and residue (g).

### 2.4. Permeability Testing

**Figure 3:**
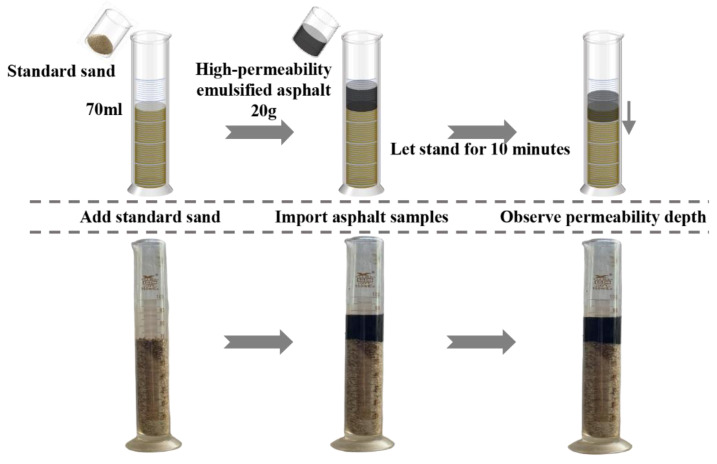
Experimental procedure of the sand cylinder method for permeability evaluation.

The permeability of the high-permeability emulsified asphalt was evaluated using the validated standard sand cylinder method [11,22]. Permeability performance was characterized using two metrics: maximum permeability depth and average permeability rate. The corresponding experimental procedure is illustrated in Figure 3.

Standard sand with a particle size range of 0.15–1.18 mm was evenly poured into a 100 mL graduated cylinder until the 70 mL mark was reached. Subsequently, 20 g of the asphalt sample was slowly introduced into the cylinder along a glass rod to avoid disturbing the sand structure. After contact with the sand, the system was left undisturbed for 10 min. The maximum permeability depth was then measured, and the average permeability rate was calculated using Equation (3).

(3) $$V_{max}=\frac{H_{max}}{t},$$

where $V_{max}$ is the maximum permeability rate (cm/min), $H_{max}$ represents the maximum permeability depth (cm), and t is the permeability duration (min), which was set to 10 min in this study.

### 2.5. Viscosity Testing

**Figure 4:**
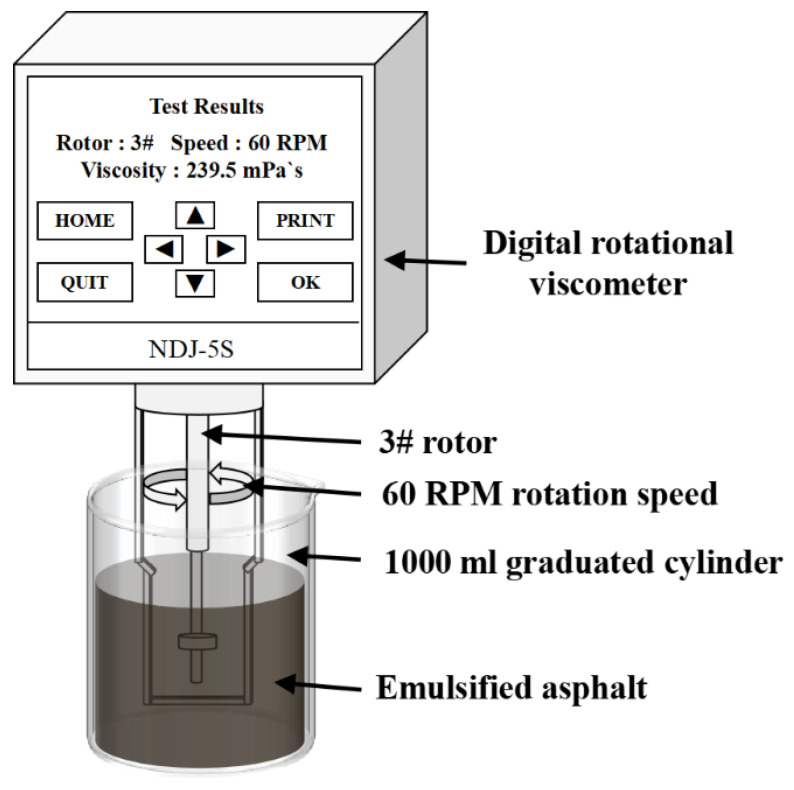
Viscosity Testing.

The viscosity of emulsified asphalt samples with various formulation ratios was measured using an NDJ-5S digital rotational viscometer. The instrument has a measurement range of 0.1–105 mPa·s and a measurement accuracy of ±1% for Newtonian fluids. All tests were conducted at ambient room temperature with rotor no. 3 provided by the manufacturer, and the rotation speed was maintained at 60 rpm. Emulsified asphalt samples were contained in a 1000 mL graduated cylinder prior to testing [23,24]. Before each measurement, the sample was transferred into the viscometer cup, ensuring that the liquid level completely submerged the rotor’s reference line. A vertical clearance of at least 10 mm was maintained between the bottom of the cup and the rotor, and a lateral clearance of no less than 15 mm was maintained between the rotor and the cup wall. The viscometer was then activated, and the viscosity value was recorded upon completion of the measurement cycle. The corresponding experimental procedure is illustrated in Figure 4.

### 2.6. Adhesion Performance Test

The adhesion of high-permeability emulsified asphalt to the aggregate surface of the base layer directly affects its bonding performance when applied as a prime coat [6,25]. Surface free energy (SFE) theory explains the asphalt-aggregate adhesion and stripping from an interfacial energy perspective, where SFE is defined as the energy required to separate a material to create a new interface [16,26]. According to the Generalized van Oss–Chaudhury (GvOC) model [26], the SFE of a solid or liquid comprises a dispersive component and a polar component. The polar component can be further subdivided into Lewis acid and Lewis base contributions, as expressed in Equation (4).

(4) $$\gamma =\gamma^{d}+\gamma^{p}=\gamma^{d}+2\sqrt{\gamma^{+}\gamma^{-}},$$

where γ is the total surface free energy of the material, $\gamma^{d}$ is the dispersive component, $\gamma^{p}$ is the polar component, $\gamma^{+}$ is the Lewis acid, and $\gamma^{-}$ is the Lewis base.

When a solid interacts with a liquid, their interfacial energy changes to achieve a solid–liquid equilibrium. This interfacial free energy can be quantified by the geometric mean of the dispersive components from both phases, combined with their polar interactions, as shown in Equation (5).

(5) $$\gamma_{sl}=\gamma_{s}+\gamma_{l}-2\sqrt{\gamma_{s}^{d}\gamma_{l}^{d}}-2\sqrt{\gamma_{s}^{p}\gamma_{l}^{p}},$$

where $\gamma_{sl}$ is the total surface free energy of the material; $\gamma_{s}$ and $\gamma_{l}$ are the total surface free energies of the solid and liquid, respectively; $\gamma_{s}^{d}$ and $\gamma_{s}^{p}$ are the dispersive and polar components of the solid surface energy, respectively; and $\gamma_{l}^{d}$ and $\gamma_{l}^{p}$ are the corresponding components of the liquid.

Young’s equation establishes a relationship between the contact angle of a test liquid on a solid surface and the surface energy of the solid, as given in Equation (6).

(6) $$\gamma_{s}=\gamma_{sl}+\gamma_{l}\cos\theta ,$$

where θ is the contact angle at the solid-liquid-vapor interface.

Substituting Equation (5) into Equation (6) allows for the derivation of Equation (7). Consequently, by measuring the contact angles (θ) of three test liquids with known SFE components on the solid, Equation (7) can be used to determine the solid’s dispersive and polar SFE components. The total SFE is then calculated using Equation (4).

(7) $$\frac{1+\cos\theta }{2}\gamma_{l}=\sqrt{\gamma_{s}^{d}\gamma_{l}^{d}}+\sqrt{\gamma_{s}^{p}\gamma_{l}^{p}}=\sqrt{\gamma_{s}^{d}\gamma_{l}^{d}}+\sqrt{\gamma_{s}^{+}\gamma_{l}^{-}}\sqrt{\gamma_{s}^{-}\gamma_{l}^{+}},$$

where $\gamma_{s}^{+}$ and $\gamma_{s}^{-}$ are Lewis acid and Lewis base of the solid, respectively, and $\gamma_{l}^{+}$ and $\gamma_{l}^{-}$ are Lewis acid and Lewis base of the liquid, respectively.

Furthermore, thermodynamic theory defines the work required to separate a homogeneous asphalt material, creating two new interfaces, as the cohesive work ($W_{a}$). This work equals twice the SFE of the asphalt ($\gamma_{a}$), as given by Equation (8).

(8) $$W_{a}=2\gamma_{a},$$

where $W_{a}$ is the cohesive work, $\gamma_{a}$ is the surface free energy of the asphalt.

In an asphalt mixture, the asphalt coats and adheres to the aggregate surface, altering the SFE of the asphalt-aggregate system. The energy required to separate the asphalt-aggregate interface under waterless conditions is termed the asphalt-aggregate adhesion work ($W_{as}$). This parameter is a key indicator of the bond performance between asphalt and aggregate. A higher adhesion work value indicates that more energy is required to debond the asphalt from the aggregate. Consequently, this signifies stronger bond strength and enhanced system stability [27]. The adhesion work is quantified by Equation (9). Its expanded form is derived by incorporating the SFE components from Equation (5).

(9) $$W_{as}=\gamma_{a}+\gamma_{s}-\gamma_{as}=2\sqrt{\gamma_{a}^{d}\gamma_{s}^{d}}+2\sqrt{\gamma_{a}^{p}\gamma_{s}^{p}}=2\sqrt{\gamma_{a}^{d}\gamma_{s}^{d}}+2\sqrt{\gamma_{a}^{+}\gamma_{s}^{-}}+2\sqrt{\gamma_{a}^{-}\gamma_{s}^{+}},$$

where $W_{as}$ is the adhesion work, $\gamma_{s}$ is the surface free energy of the aggregate, and $\gamma_{as}$ is the interfacial free energy between asphalt and aggregate.

**Figure 5:**
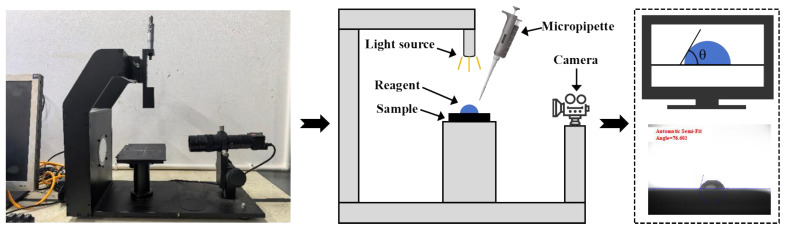
Contact angle measurement apparatus and testing principle.

In this study, the contact angles of twenty high-permeability emulsified asphalt evaporation residue samples were measured using an SL-200 optical contact angle and interfacial tension analyzer (Shanghai Solon Information Technology Co., Ltd., Shanghai, China). The instrument comprises three main systems: a light source, a video capture system, and an analysis system. The complete experimental setup is illustrated in Figure 5. The measurement principle involves (1) capturing the liquid–sample contact process with a high-resolution camera, (2) image stabilization using analysis software, and (3) static contact angle calculation via the circle-fitting method. Detailed experimental procedures follow the methodology described in [26].

Distilled water, glycerol, and formamide were selected as probe liquids. Contact angles were measured at 25 °C using the sessile drop method. Each measurement was repeated five times per sample, and the average value was calculated to minimize experimental variation. The surface energy components for the three probe liquids and the aggregate are summarized in Table 6.

## 3. Result and Discussion

### 3.1. Fundamental Physical Performances

**Figure 6:**
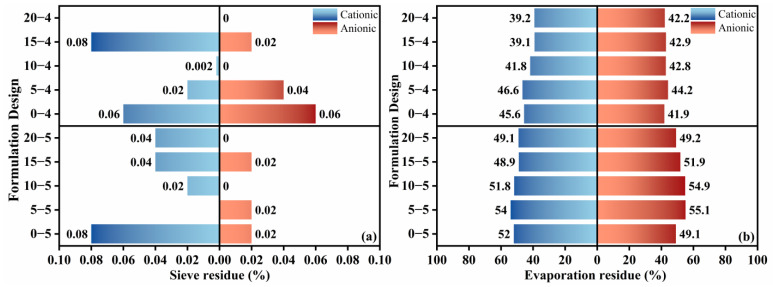
Test results for high-permeability emulsified asphalt. (**a**) Sieve residue and (**b**) evaporation residue.

The fundamental physical performances of the high-permeability emulsified asphalt formulations were evaluated through residue on sieve, evaporation residue content, and the penetration, softening point, and ductility of the evaporation residue. The residue on the sieve test examines particle dispersion and uniformity within the emulsion, while the evaporation residue reflects the mass of usable asphalt, with the results presented in Figure 6. In the sieve residue test, anionic emulsions exhibited markedly lower residue than their cationic counterparts, indicating that anionic emulsifiers promote more complete mixing and emulsification of asphalt with the soap solution. With increasing kerosene dosage, residue on the sieve generally declined, and higher diluted asphalt content yielded better emulsification quality. Notably, adding a small amount of kerosene increased the evaporation residue content, whereas further increases in kerosene led to a decrease in evaporation residue, consistent with previous findings that kerosene addition can reduce the evaporation residue of emulsified asphalt [14]. Increasing the diluted asphalt content raised the evaporation residue content, in agreement with prior studies [13], while the effect of emulsifier ionic type on evaporation residue was comparatively limited. Because the evaporation residue represents the effective material in emulsified asphalt, introducing a small kerosene dosage can reduce cost while maintaining effectiveness. To ensure construction quality and enhance emulsification, anionic emulsifiers are recommended as the first choice.

**Figure 7:**
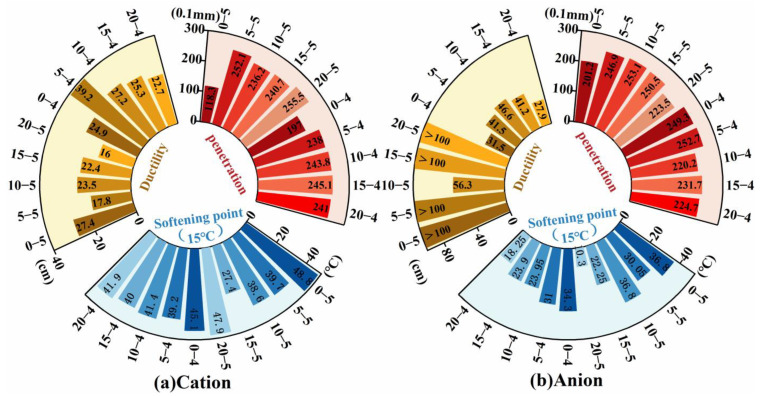
Test results for penetration, elongation, and softening point of high-permeability emulsified asphalt evaporation residue. (**a**) Cationic and (**b**) anionic.

The penetration, softening point, and ductility of the evaporation residue were then assessed (Figure 7). Overall, the softening point and ductility were strongly influenced by the emulsifier ionic type: cationic emulsions produced higher softening points, whereas anionic emulsions delivered greater ductility. Adding kerosene increased penetration, reduced the softening point, and caused ductility to first rise and then fall. Ductility decreased as the diluted asphalt content decreased. The results indicate that cationic emulsifiers can effectively improve high-temperature stability, while anionic emulsifiers substantially enhance low-temperature extensibility and crack resistance, in several anionic formulations, ductility exceeded 100 cm. Consistent with earlier reports [1,14], kerosene addition weakened deformation resistance and high-temperature stability by lowering asphalt viscosity and inter-particle interactions. Importantly, reducing the diluted asphalt content partially mitigated kerosene-induced reductions in deformation resistance. The influence of diluted asphalt content on low-temperature performance differed by ionic type: for cationic emulsions, plastic deformation capacity declined with decreasing diluted asphalt content, whereas the opposite trend was observed for anionic emulsions. The different low-temperature performance of cationic and anionic systems with varying liquefied bitumen contents suggests distinct demulsification mechanisms and residual emulsifier effects [29,30]. However, this interpretation requires validation through direct microstructural characterization using techniques such as SEM or AFM, which will be addressed in subsequent studies. These observations suggest a potential interaction between emulsifier ionic type and diluted asphalt content for the ductility index, which is further explored via grey relational analysis. Given that emulsifier ionic type, kerosene dosage, and diluted asphalt content can exert competing effects on deformation resistance, high-temperature stability, and low-temperature performance, selection should be tailored to service requirements. For enhanced low-temperature extensibility and crack resistance, an anionic emulsifier with higher diluted asphalt content and a modest kerosene dosage is preferred. Where high-temperature stability is critical, a cationic emulsifier should be prioritized, and kerosene addition avoided.

### 3.2. Permeability Analysis

**Figure 8:**
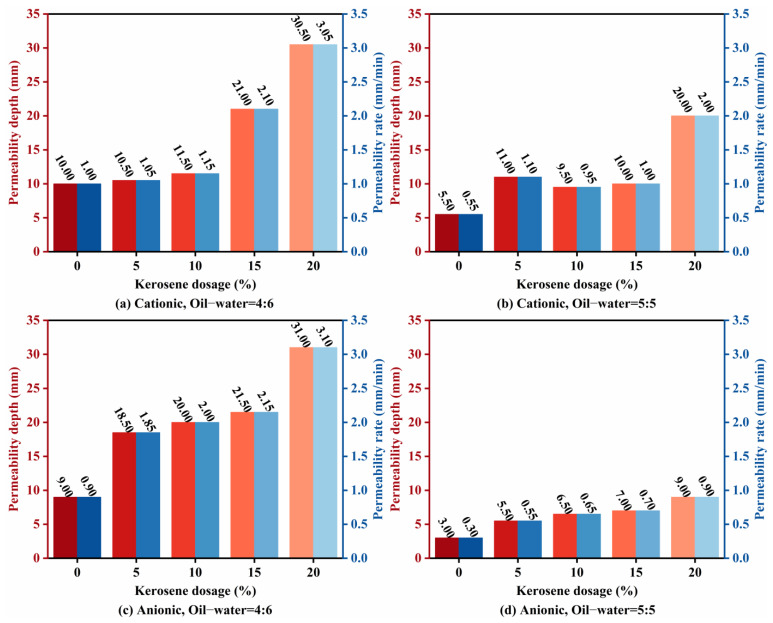
High-permeability emulsified asphalt permeability performance test results.

Permeability was assessed using the validated “standard sand-cylinder” method, and the results are shown in Figure 8. The effect of emulsifier ionic type on permeability depended on the diluted asphalt content: at a diluted asphalt content of 40% (oil-water ratio 4:6), anionic emulsions performed better, while at 50% (5:5), cationic emulsions were superior. Both adding kerosene and reducing the diluted asphalt content significantly improved permeability, consistent with prior work [13,31]. At an oil-water ratio of 4:6 with 20% kerosene, the average permeability rate exceeded 3 cm/min, an effect sometimes described as “instantaneous permeability” [14]. Relative to undiluted high-permeability emulsified asphalt, adding 10% kerosene increased the permeability depth of the four groups in Figure 7 by 15%, 72.73%, 122.22%, and 116.67%, respectively, indicating a stronger kerosene effect for anionic systems. Increasing the diluted asphalt content substantially reduced permeability, likely because the reduced soap solution fraction increases surface tension and weakens wetting, furthermore, the higher droplet density at higher oil-water ratios promotes faster demulsification and earlier film formation, both of which suppress permeability. As highlighted in Figure 8b, the “increase-decrease-increase” trend in evaporation residue content (Figure 6) parallels the permeability evolution, suggesting a linkage examined later via grey relational analysis. In practice, adopting a lower diluted asphalt content and/or introducing a small kerosene dosage can better ensure that permeability depth into the base.

### 3.3. Viscosity Analysis

**Figure 9:**
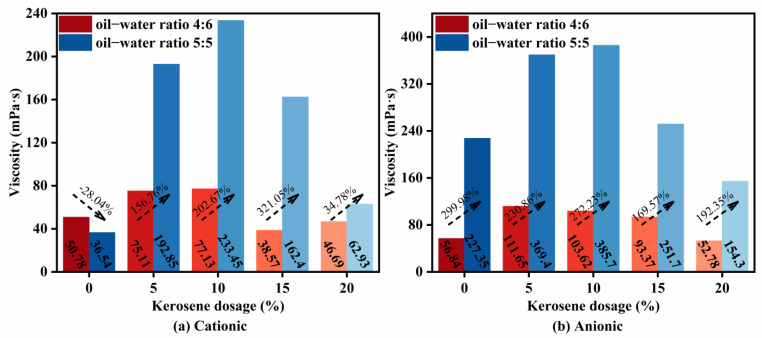
High-permeability emulsified asphalt rotational viscosity test results. (**a**) Cationic and (**b**) anionic.

Rotational viscosity testing (Figure 9) was used to evaluate flow behavior across formulations. Anionic emulsions exhibited higher viscosity than cationic emulsions, and viscosity increased with diluted asphalt content, with average increases of 137.44% (cationic) and 233% (anionic). This underscores a stronger sensitivity of flow behavior to diluted asphalt content in anionic systems. Across all formulations, viscosity rose at low kerosene dosage and then decreased at higher dosage, mirroring the trend in evaporation residue content reported in Section 3.1 and aligning with prior findings that higher evaporation residue generally corresponds to higher viscosity [32]. The viscosity trends were broadly consistent with permeability (Figure 8): lower viscosity was associated with better permeability. From a formulation design standpoint, lower diluted asphalt content produced smaller viscosity fluctuations, yielding more robust and controllable flowability during construction.

### 3.4. Adhesion Performance Analysis

**Figure 10:**
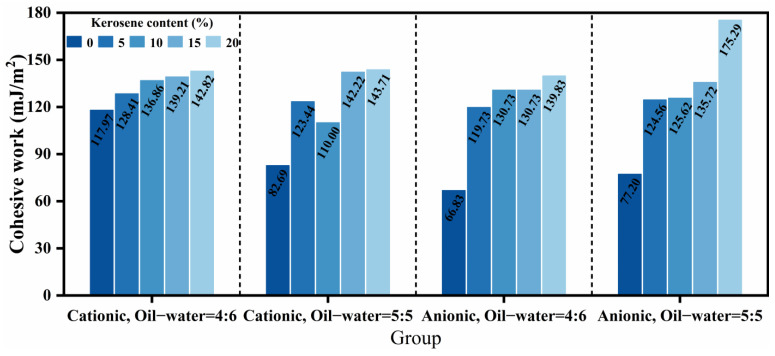
Cohesive work of high-permeability emulsified asphalt samples.

The cohesive work of asphalt serves as a critical indicator for assessing its intrinsic bonding capacity. A higher cohesive work value indicates reduced susceptibility to cohesive failure under stable conditions, conferring enhanced resistance to deformation and relative displacement caused by external forces [27]. The cohesive work values were calculated from contact angle measurements of the twenty high-permeability emulsified asphalt formulations, as presented in Figure 10. Overall, emulsifier ionic type and diluted asphalt content exhibited limited effects on cohesive work, while increasing kerosene dosage progressively enhanced it. This enhancement may stem from alterations in the surface composition of emulsified asphalt during demulsification and evaporation processes upon kerosene incorporation, ultimately manifested in the cohesive work values [33]. Specifically, the addition of 20% kerosene increased the cohesive work by 21.06%, 73.79%, 109.23%, and 127.05% across the four respective formulations when compared with undiluted asphalt, demonstrating a more pronounced enhancement effect in anionic systems and formulations with higher diluted asphalt content. Furthermore, the variation trends in cohesive work across formulations correlated well with the permeability characteristics discussed in Section 3.2, suggesting a potential fundamental relationship. In practical applications, kerosene dosage represents the primary adjustable parameter for modulating cohesive work to achieve optimal bonding performance.

**Figure 11:**
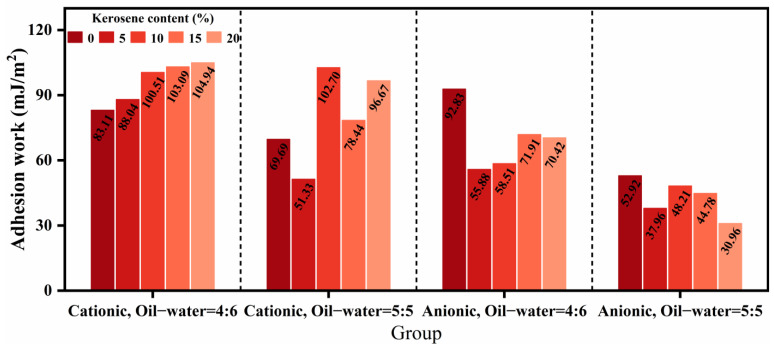
Adhesion work of high-permeability emulsified asphalt with limestone.

Adhesion work directly characterizes the interfacial bonding performance between emulsified asphalt and aggregate, correlating closely with key macroscopic properties including moisture resistance, stripping resistance, and long-term durability [16]. The adhesion work values for the twenty formulations were calculated based on surface free energy theory, with results presented in Figure 11. In general, cationic emulsified asphalt demonstrated higher adhesion work, consistent with established mechanisms wherein cationic emulsifiers promote stronger interactions with negatively charged aggregate surfaces through combined electrostatic and polar effects, thereby enhancing interfacial bonding [34]. Furthermore, increased diluted asphalt content reduced adhesion work, potentially resulting from the way in which higher soap solution content diminishes interfacial tension and enhances wettability, thereby promoting asphalt spreading across aggregate surfaces [6,35]. Additionally, the effect of kerosene dosage on adhesion work exhibited significant dependence on emulsifier ionic type. In cationic systems, adhesion work increased progressively with kerosene dosage, while an inverse relationship was observed in anionic systems. Notably, At the 4:6 oil-water ratio, variations in adhesion work with emulsifier type correlated inversely with permeability performance: higher adhesion work values generally corresponded to reduced permeability. This inverse correlation indicates that enhanced adhesion work corresponds to improved interfacial stability and strengthened asphalt-aggregate bonding, consistent with previous investigations [26]. In engineering practice, emulsifier selection should be application-specific rather than generalized. Anionic systems are particularly suitable for applications requiring enhanced low-temperature crack resistance, while cationic systems are preferred for scenarios demanding superior interfacial bonding performance.

### 3.5. Grey Relational Analysis

Grey relational analysis (GRA) is well suited to systems characterized by limited samples and uncertain information, it can rank the relative importance of factors and quantify their effects on system performance with modest computational burden and minimal assumptions. Based on grey theory [36,37,38], a GRA model was established using Python 3.7 software to relate composition variables, kerosene dosage (K), emulsifier ionic type (I), and diluted asphalt content (R), to performance metrics including residue on sieve (SR), evaporation residue (ER), penetration (P), softening point (SP), ductility (D), permeability depth (PD), viscosity (V), cohesive work ($W_{a}$), and adhesion work ($W_{as}$). A summary of the experimental results used for analysis is provided in Table 7.

For a system characteristic series that includes multiple related factor series, the pointwise difference between each related factor curve and the system–characteristic curve can be expressed by Equation (10) [38].

(10) $$\xi_{i}\left(k\right)=\frac{\underset{i}{\min}\;\underset{k}{\min}\left|x_{0}-x_{i}\left(k\right)\right|+\rho \;\underset{i}{\max}\;\underset{k}{\max}\left|x_{0}-x_{i}\left(k\right)\right|}{\left|x_{0}-x_{i}\left(k\right)\right|+\rho \;\underset{i}{\max}\;\underset{k}{\max}\left|x_{0}-x_{i}\left(k\right)\right|},$$

where $\xi_{i}\left(k\right)$ represents the relative difference between the kth relevant factor curve $x_{i}$ and the system characteristic curve $x_{0}$, which can be interpreted as the grey relational coefficient of $x_{i}$ relative to $x_{0}$ at point k. ρ is the resolution coefficient, typically chosen between 0 and 1, with a common value of 0.5, while $\underset{i}{\min}\;\underset{k}{\min}\left|x_{0}-x_{i}\left(k\right)\right|$ is referred to as the minimum difference between the two levels and $\underset{k}{\min}\left|x_{0}-x_{i}\left(k\right)\right|$ represents “selecting the minimum value among k points.” The second level is “selecting the minimum among all i,” and $\underset{i}{\max}\;\underset{k}{\max}\left|x_{0}-x_{i}\left(k\right)\right|$ is the maximum difference between the two levels.

Although the foregoing steps yield the two-level minimum and maximum differences, they are not sufficient for the direct computation of the grey relational coefficient. This is because the series used in grey relational analysis must share commensurate dimensions, when the dimensions are inconsistent, the data must be standardized to avoid biasing the analysis. In this study, the min-max normalization method was employed to normalize the raw data, linearly mapping them to the [0,1] interval and thereby eliminating dimensional discrepancies that could affect the grey relational analysis, the transformation is given by Equation (11).

(11) $$X_{n}=\frac{X-X_{min}}{X_{max}-X_{min}},$$

where $X_{n}$ represents the standardized value, X is the original value, and $X_{max}$ and $X_{min}$ are the maximum and minimum values in the feature column, respectively.

**Figure 12:**
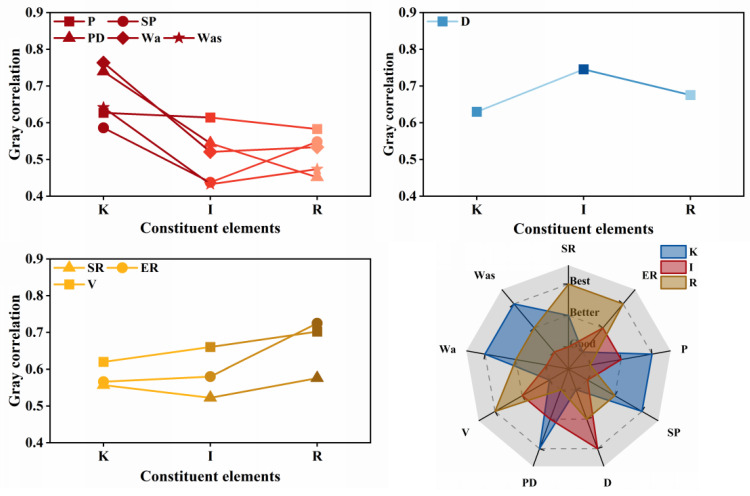
Grey relational analysis of different variables on performance.

In this study, for the emulsifier ionic type, the normalized values were retained as 0 and 1 to preserve the categorical information. After normalization, the geometric similarity among the series can be compared fairly, laying the foundation for subsequent grey relational analysis. The data were first normalized using Equation (11), and then the grey relational coefficients between the formulation design factors of high-permeability emulsified asphalt and each performance index were computed via Equation (10), yielding multiple coefficient sequences. Because the number of grey relational coefficients is large and their behavioral information is dispersed, direct visual comparison is inconvenient, therefore, the averaging method is commonly adopted to obtain the grey relational grades for cross-factor comparison, which are calculated by Equation (12).

(12) $$r_{i}=\frac{1}{N}\sum_{k=1}^{N}\xi_{i}\left(k\right),$$

where $r_{i}$ denotes the grey relational grade of each performance index with respect to the formulation design factors and N is the number of data points in a single series—in this study, N = 20. Accordingly, the grey relational grades of kerosene dosage, emulsifier ionic type, and diluted asphalt content (oil–water ratio) with respect to residue on sieve, evaporation residue, penetration, softening point, ductility, permeability depth, viscosity, cohesive work, and adhesion work are shown in Figure 12. Among these, kerosene dosage exerted the most significant influence on penetration (grade = 0.6270), softening point (0.5860), permeability depth (0.7398), cohesive work (0.7635), and adhesion work (0.6413); emulsifier ionic type most strongly affected ductility (0.7457); and diluted asphalt content (oil–water ratio) dominated residue on sieve (0.6519), evaporation residue (0.7247), and viscosity (0.7021). These results indicate that, when optimizing the mix of high-permeability emulsified asphalt, kerosene dosage should be prioritized to tune deformation resistance, high-temperature stability, permeability, and adhesion; that improvement of low-temperature performance should focus on the selection of emulsifier ionic type, and emulsification quality; and that flowability should be controlled primarily by adjusting the diluted asphalt content (oil-water ratio) to achieve the desired outcome.

**Figure 13:**
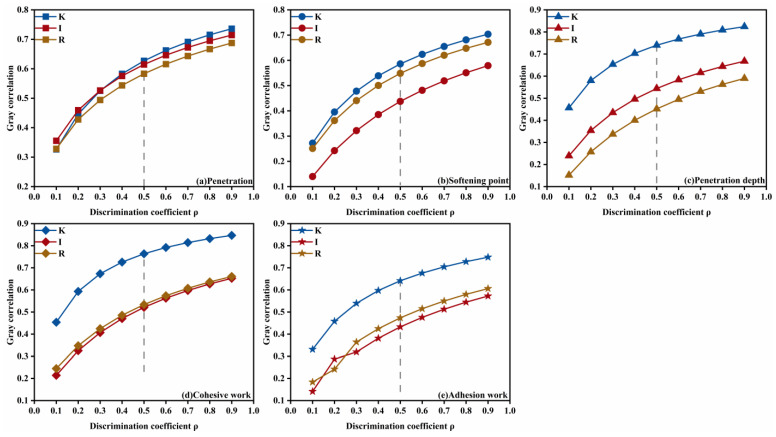
Sensitivity of kerosene-dosage dominated indicators to changes in the resolution coefficient.

Meanwhile, because different choices of the resolution coefficient can influence the resulting grey relational grades, this study conducted sensitivity analyses for all performance indices with *ρ* from 0.1 to 0.9 and grouped the indices according to the most significant governing factor in the formulation design. For the kerosene-dosage dominated indicators, the variation of the grey relational grade with *ρ* is shown in Figure 13. Kerosene dosage exerted the most pronounced influence on penetration, softening point, permeability depth, cohesive work, and adhesion work, and this prominence did not change as *ρ* varied. However, for penetration, when *ρ* < 0.5, the influence of the emulsifier ionic type exceeded that of kerosene dosage, with the two grades being close in magnitude, in line with a similar phenomenon that was observed for the softening point. These findings suggest that the high-temperature properties and thermal stability of high-permeability emulsified asphalt are likely governed by interacting factors.

**Figure 14:**
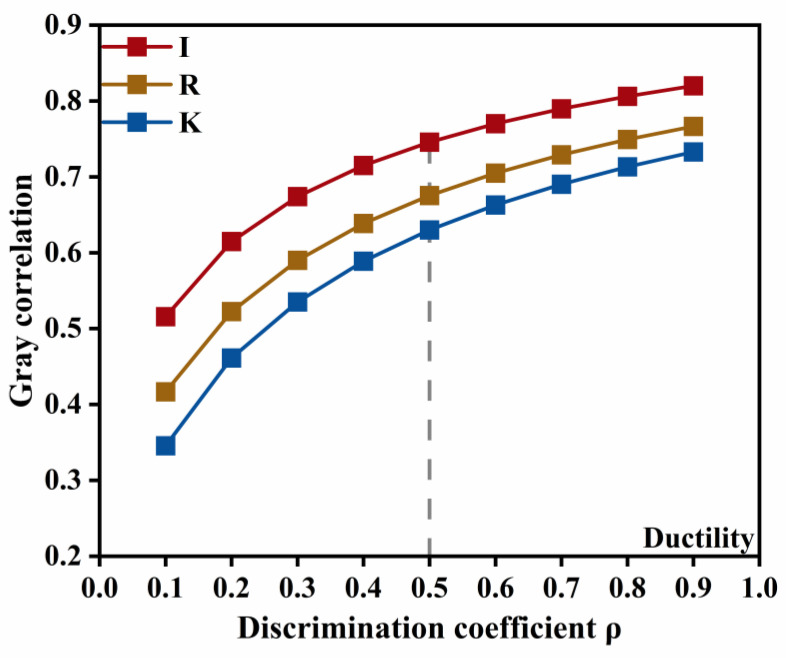
Sensitivity of emulsifier ionic type dominated indicators to changes in the resolution coefficient.

For the ionic type of dominated indicators, the variation of the grey relational grade with the resolution coefficient is shown in Figure 14. The prominence of the emulsifier ionic type for ductility does not vary with changes in the resolution coefficient, and its influence on ductility is markedly stronger than that of the other two factors. This indicates that the emulsifier ionic type is the primary determinant of low-temperature performance, and that interaction effects may be neglected in this case.

**Figure 15:**
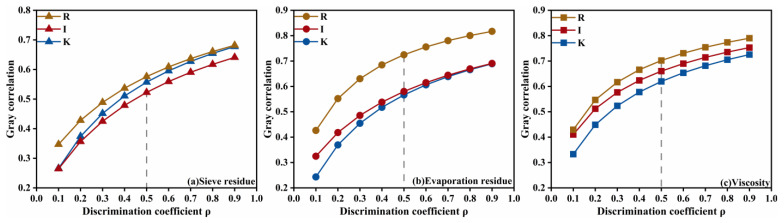
Sensitivity of diluted asphalt content dominated indicators to changes in the resolution coefficient.

For the diluted asphalt content (oil-water ratio) dominated indicators, the variation of the grey relational grade with the resolution coefficient is shown in Figure 15. The prominence of diluted asphalt content for residue on sieve, evaporation residue, and viscosity does not change with the resolution coefficient, and its influence on residue on sieve and evaporation residue is markedly stronger than that of the other two factors. This indicates that diluted asphalt content occupies a dominant role in governing the emulsification characteristics of high-permeability emulsified asphalt, whereas its grey relational grade for viscosity is not significantly higher than those of the other factors-implying that viscosity is likely subject to multi-factor interactions.

Considering the variation of grey relational grades with the resolution coefficient across the three factor-dominated groups, adopting *ρ* = 0.5 for the grey relational analysis of the effects of formulation design factors on the performance of high-permeability emulsified asphalt is appropriate, and the resulting ranking of influencing factors is credible. Moreover, because the grey relational grades of certain factors are close in magnitude, an analysis of interaction effects among factors is subsequently undertaken to further elucidate this phenomenon.

Additionally, prior studies have reported close relationships between the evaporation residue content and multiple properties of emulsified asphalt [32,39], though the specific associations remain unclear. Therefore, this study employed grey relational analysis to quantify the influence of evaporation residue content in high-permeability emulsified asphalt on its fundamental physical properties, permeability, and viscosity. The calculated order of influence was as follows: viscosity (grade = 0.7284) > ductility (0.6837) > residue on sieve (0.6664) > cohesive work (0.6389) > softening point (0.6187) > penetration (0.6132) > adhesion work > permeability depth (0.5725). These results indicate that evaporation residue content has a greater impact on flowability, low-temperature performance, and emulsification quality, and a comparatively smaller effect on deformation resistance, high-temperature stability, adhesion, and permeability, consistent with the conclusion in Section 3 stating that the magnitude of evaporation residue content determines the amount of effective material and thereby governs overall performance.

Although single factor grey relational analysis clearly reveals the dominant influences and determinant parameters of different formulation design factors on the performance of high-permeability emulsified asphalt, it neglects nonlinear interaction effects among factors. In practice, the performance of high-permeability emulsified asphalt is not governed by a single factor mechanism. For example, within an emulsified asphalt system, kerosene can improve permeability by disrupting the long molecular chains in the asphalt components, while the emulsifier enhances permeability by reducing the interfacial tension at the asphalt/water boundary, moreover, different emulsifier ionic types yield distinct outcomes. Acting together, these factors jointly regulate permeability. Therefore, this study extends the grey relational model by introducing second order interaction terms—kerosene dosage and emulsifier ionic type (K&I), kerosene dosage and diluted asphalt content (K&R), and emulsifier ionic type and diluted asphalt content (I&R)—as well as a third order interaction term—kerosene dosage and emulsifier ionic type and diluted asphalt content (K&I&R), with the aim of quantifying the synergistic effects among formulation design factors and identifying critical thresholds in the formulation design. Specifically, the product method multiplies the normalized values of the two or three participating factors, when all factors are simultaneously at high/low levels, the product is markedly amplified, which facilitates discrimination of interaction effects. The constructions of these interaction terms are given by Equation (13).

(13) $$X_{interact}=X_{i}\times X_{j},$$

where $X_{interact}$ is the value of the interaction term, and $X_{i}$ and $X_{j}$ are the normalized values of different factors. We substitute the calculated $X_{interact}$ into the above grey relational analysis steps to obtain the grey relational of the interaction term.

**Figure 16:**
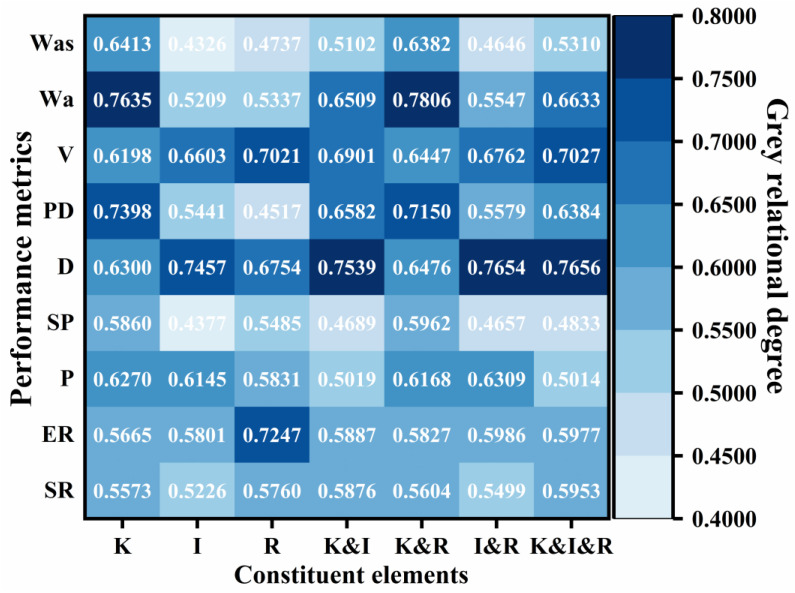
Multifactorial interaction effects analysis.

Building on the foregoing theory and equations, the interaction effects in multi-factor grey relational analysis were elucidated, and the computed results are shown in Figure 16. The interaction between kerosene dosage and diluted asphalt content (K&R) exhibited pronounced influences on the softening point (grade = 0.5962), permeability depth (0.7150), cohesive work (0.7806), and adhesion work (0.6382). The interaction between emulsifier ionic type and diluted asphalt content (I&R) showed a significant cooperative effect on penetration (0.6309). The third order interaction (K&I&R) had strong effects on residue on sieve (0.5953), ductility (0.7656), and viscosity (0.7027). These findings indicate that high-temperature stability, permeability, and adhesion should be tuned with priority given to the K&R interaction; that deformation resistance should focus on the I&R interaction; and that emulsification characteristics, low-temperature performance, and flowability should account for multi-factor coordination. This clarifies the mechanisms by which formulation design adjustments improve performance, avoids the limitations and experimental brittleness of single-factor optimization, and provides a theoretical basis for subsequent performance-oriented formulation design optimization.

## 4. Conclusions

This study quantitatively assessed the effects of emulsifier ionic type, kerosene dosage, and oil-water ratio on the performance of high-permeability emulsified asphalt. Based on comprehensive experimental evaluation and grey relational analysis, the following conclusions were drawn:

(1) Anionic emulsifiers significantly enhanced permeability, viscosity, and low-temperature ductility, indicating superior overall performance compared with cationic systems.
(2) Kerosene addition markedly improved permeability and viscosity at low dosage (<10%), but adversely affected high- and low-temperature stability when overdosed.
(3) Reducing the diluted asphalt content improved permeability, adhesion performance, and mitigated the thermal degradation caused by kerosene, albeit with minor trade-offs in viscosity.
(4) Grey relational analysis revealed that kerosene dosage was the dominant factor influencing permeability, softening point, and adhesion performance, emulsifier ionic type primarily governed low-temperature flexibility, and oil-water ratio critically affected emulsification efficiency and viscosity.

This study comprehensively investigated the effects of composition on the performance characteristics of high-permeability emulsified asphalt. However, the present conclusions are limited to statistically significant macroscale correlations due to the absence of multi-scale experimental validation. Future research should incorporate multi-scale experimental approaches, with emphasis on microstructural characterization, including analysis of demulsification mechanisms, nanoscale heterogeneity, rheological behavior (e.g., SEM and AFM), and direct mechanical evaluation using interlayer shear and peel tests on representative substrates. Additionally, the development of a formal multi-criteria optimization framework is recommended, where prime coat effectiveness would be evaluated based on simultaneous satisfaction of key performance requirements including permeability, interfacial bonding, constructability, and thermal/low-temperature performance. The application of project- and climate-specific weighting schemes combined with advanced optimization techniques is suggested to identify optimal compromise solutions among competing criteria. These proposed approaches would facilitate more rational material design optimization and ensure enhanced long-term durability under diverse service conditions.

## Figures and Tables

**Table 1:** Basic properties of matrix asphalt.

Index		Gaofu70#	Requirement	Test Method [17]
Penetration (25 °C, 100 g, 5 s), 0.1 mm		68.13	60~80	T0604
Soften point, °C		49.1	≥46	T0606
Ductility (15 °C, 5 cm/min), cm		21.0	≥20	T0605
Dynamic viscosity, Pa·s		230	≥180	T0620
Penetration (25 °C, 100 g, 5 s), 0.1 mm		68.13	60~80	T0604
RTFOT	Mass variation, %	0.182	≤±0.8	T0610
	Residual penetration ratio, %	62.1	≥61	

**Table 2:** Basic properties of the emulsifiers.

Index	CTAB	SDBS	OP-10
Structural formula	C19H42BrN	C18H29NaO3S	-
Relative molecular mass	364.45	348.48	-
Appearance	White crystals	-	Colorless or slightly yellow
Content, %	≥99.0	≥90.0	≥98.0
pH value	5.0~7.0	-	6.0~8.0
Turbidity point, °C	-	-	60-68
Fe, %	≤0.001	-	-
Heavy metal (Pb), %	≤0.0005	-	-
Water content, %	≤0.5	4.0	-
Combustion burned residues (sulfates), %	≤0.1	-	-
Ethanol solubility test	qualified	-	-
Water solubility test	qualified	-	-
Sodium sulfate	-	6.0	-
Heavy metal (Pb), %	≤0.0005	-	-

**Table 3:** Basic properties of penetrant.

Index	JFC-E
Structural formula	RO-(CH_2_CH_2_O)n-H, R = isooctyl
Appearance	Light yellow transparent or milky white
Active ingredient, %	≥99.5
Turbidity point, °C	64~68
pH value	6.8~7.2

**Table 4:** Formulation design of high-permeability emulsified asphalt.

No.	Group	Matrix Asphalt:Kerosene (by Mass)	Emulsifier Type	Oil–Water Ratio
1	C-0-5	Matrix asphalt:Kerosene = 100:0	Cationic	5:5
2	C-5-5	Matrix asphalt:Kerosene = 95:5		5:5
3	C-10-5	Matrix asphalt:Kerosene = 90:10		5:5
4	C-15-5	Matrix asphalt:Kerosene = 85:15		5:5
5	C-20-5	Matrix asphalt:Kerosene = 80:20		5:5
6	C-0-4	Matrix asphalt:Kerosene = 100:0		4:6
7	C-5-4	Matrix asphalt:Kerosene = 95:5		4:6
8	C-10-4	Matrix asphalt:Kerosene = 90:10		4:6
9	C-15-4	Matrix asphalt:Kerosene = 85:15		4:6
10	C-20-4	Matrix asphalt:Kerosene = 80:20		4:6
11	S-0-5	Matrix asphalt:Kerosene = 100:0	Anionic	5:5
12	S-5-5	Matrix asphalt:Kerosene = 95:5		5:5
13	S-10-5	Matrix asphalt:Kerosene = 90:10		5:5
14	S-15-5	Matrix asphalt:Kerosene = 85:15		5:5
15	S-20-5	Matrix asphalt:Kerosene = 80:20		5:5
16	S-0-4	Matrix asphalt:Kerosene = 100:0		4:6
17	S-5-4	Matrix asphalt:Kerosene = 95:5		4:6
18	S-10-4	Matrix asphalt:Kerosene = 90:10		4:6
19	S-15-4	Matrix asphalt:Kerosene = 85:15		4:6
20	S-20-4	Matrix asphalt:Kerosene = 80:20		4:6

**Table 5:** High permeability emulsified asphalt soap liquid ratio.

Type	Primary Emulsifier	Co-Emulsifier	Penetrant	pH-Adjusting Agent	pH Range
Cationic	CTAB, 4.0%	OP-10, 2.5%	JFC-E, 2.5%	HCl, 1.00%	2–4
Anionic	SDBS, 2.0%			CaCl_2_, 1.25%	10–12

**Table 6:** Surface free energy components of probe liquids and aggregate (25 °C).

Probe Liquid/Aggregate	$\gamma_{l}$	$\gamma_{l}^{d}$	$\gamma_{l}^{p}$	$\gamma_{l}^{+}$	$\gamma_{l}^{-}$
Distilled water	72.8	21.8	51	25.5	25.5
Glycerol	64	34	30	3.92	57.4
Formamide	57.9	38.9	19	2.28	39.6
Limestone [28]	145.11	143.22	1.89	0.0023	393.68

**Table 7:** Summary of test results for high-permeability emulsified asphalt.

Group	SR, %	ER, %	P, 0.1 mm	SP, °C	D, cm	PD, mm	V, mPa·s	$W_{a}$, mJ/m^2^	$W_{as}$, mJ/m^2^
C-0-5	0.08	52	118.3	48.8	27.4	5.5	36.54	82.69	69.69
C-5-5	0	54	252.1	39.7	17.8	11	192.85	123.44	51.33
C-10-5	0.02	51.8	236.2	38.6	23.5	9.5	233.45	110.00	102.70
C-15-5	0.04	48.9	240.7	27.4	22.4	10	162.4	142.22	78.44
C-20-5	0.04	49.1	255.5	47.9	16	20	62.93	143.71	96.67
C-0-4	0.06	49.1	201.2	36.8	100	3	227.35	117.97	83.11
C-5-4	0.02	55.1	246.9	30.05	100	5.5	369.4	128.41	88.04
C-10-4	0.002	54.9	253.1	36.8	56.3	6.5	385.7	136.86	100.51
C-15-4	0.08	51.9	250.5	22.25	100	7	251.7	139.21	103.09
C-20-4	0	49.2	223.5	10.3	100	9	154.3	142.82	104.94
S-0-5	0.02	45.6	197	45.1	24.9	10	50.78	77.20	52.92
S-5-5	0.02	46.6	238	39.2	39.2	10.5	75.11	124.56	37.96
S-10-5	0	41.8	243.8	41.4	27.2	11.5	77.13	125.62	48.21
S-15-5	0.02	39.1	245.1	40	25.3	21	38.57	135.72	44.78
S-20-5	0	39.2	241	41.9	22.7	30.5	46.69	175.29	30.96
S-0-4	0.06	41.9	249.3	34.3	31.5	9	56.84	66.83	92.83
S-5-4	0.04	44.2	252.7	31	41.5	18.5	111.65	119.73	55.88
S-10-4	0	42.8	220.2	23.95	46.6	20	103.62	130.73	58.51
S-15-4	0.02	42.9	231.7	23.9	41.2	21.5	93.37	130.73	71.91
S-20-4	0	42.2	224.7	18.25	27.9	31	52.78	139.83	70.42

## Data Availability

The original contributions presented in this study are included in the article. Further inquiries can be directed to the corresponding authors.
